# Characterization of Local and Systemic Impact of Whitefly (*Bemisia tabaci*) Feeding and Whitefly-Transmitted Tomato Mottle Virus Infection on Tomato Leaves by Comprehensive Proteomics

**DOI:** 10.3390/ijms21197241

**Published:** 2020-09-30

**Authors:** Aaron J. Ogden, Wardatou Boukari, Alba Nava, Natalia Lucinda, Garry Sunter, Wayne R. Curtis, Joshua N. Adkins, Jane E. Polston

**Affiliations:** 1 Earth and Biological Sciences Directorate, Pacific Northwest National Laboratories, Richland, WA 99354, USA; Joshua.Adkins@pnnl.gov; 2 Department of Plant Pathology, University of Florida, Gainesville, FL 32611, USA; batouka14@ufl.edu; 3 Department of Biology, South Texas Center for Emerging Infectious Disease, University of Texas at San Antonio, San Antonio, TX 78249, USA; alba.navafereira@utsa.edu (A.N.); natalia.lucindap@utsa.edu (N.L.); Garry.Sunter@utsa.edu (G.S.); 4 Department of Chemical Engineering, The Pennsylvania State University, University Park, PA 16802, USA; Wrc2@psu.edu

**Keywords:** tomato, tomato mottle virus, RNA directed DNA methylation, transcriptional gene silencing, Begomovirus, Geminivirus, *Bemisia tabaci*, whitefly, tomato, pathogenesis, proteomics

## Abstract

Tomato mottle virus (ToMoV) is a single-stranded DNA (ssDNA) begomovirus transmitted to solanaceous crops by the whitefly species complex (*Bemisia tabaci*), causing stunted growth, leaf mottling, and reduced yield. Using a genetic repertoire of seven genes, ToMoV pathogenesis includes the manipulation of multiple plant biological processes to circumvent antiviral defenses. To further understand the effects of whitefly feeding and whitefly-transmitted ToMoV infection on tomato plants (*Solanum lycopersicum* ‘Florida Lanai’), we generated comprehensive protein profiles of leaves subjected to feeding by either viruliferous whiteflies harboring ToMoV, or non-viruliferous whiteflies, or a no-feeding control. The effects of whitefly feeding and ToMoV infection were measured both locally and systemically by sampling either a mature leaf directly from the site of clip-cage confined whitefly feeding, or from a newly formed leaf 10 days post feeding (dpf). At 3 dpf, tomato’s response to ToMoV included proteins associated with translation initiation and elongation as well as plasmodesmata dynamics. In contrast, systemic impacts of ToMoV on younger leaves 10 dpf were more pronounced and included a virus-specific change in plant proteins associated with mRNA maturation and export, RNA-dependent DNA methylation, and other antiviral plant processes. Our analysis supports previous findings and provides novel insight into tomato’s local and systemic response to whitefly feeding and ToMoV infection.

## 1. Introduction

The majority of plant-infecting viruses have RNA-based genomes, suggesting plants have evolved effective mechanisms to defend against DNA viruses [1]. One exception to this evolutionary trend, the *Geminiviridae*, are the largest family of plant DNA viruses that currently include nine genera [2,3]. The largest genus [4], *Begomovirus*, consists of viruses with small circular single-stranded DNA (ssDNA) genomes that are mono- or bi-partite (referred to as DNA A and DNA B), and some are found associated with additional alpha and beta ssDNA satellites [5]. In most cases, the proteins encoded by DNA A are essential for replication, transmission, and suppressing host immunity, while DNA B encodes proteins associated with virus movement [6]. During begomovirus infection, the viral ssDNA genome is converted in the nucleus to double-stranded (ds) DNA, which then associates with plant histones forming minichromosomes resembling host chromatin. These dsDNA–histone intermediates act as templates for transcription and rolling circle replication, both of which are impacted by the accessibility of viral DNA to host proteins (i.e., chromatin density). Perhaps the most astonishing feature of the bipartite begomoviruses is the size of their genome, which consists of only four to five open reading frames (ORFs) in DNA A and 2 in DNA B [1]. Indeed, the begomoviruses are considered paragons of genetic efficiency capable of causing tremendous agricultural losses [7] with a maximum of seven genes, suggesting that their gene products are multifunctional [8].

Despite their reduced coding capacity relative to other viruses, begomoviruses effectively manipulate multiple critical plant biological processes including plant hormone signaling pathways, cell cycle progression [9,10], DNA replication [11], mRNA maturation and export [12,13], translation [14], and the epigenetic regulation of gene expression [15,16,17]. Numerous observations have been made that provide insight into how begomoviruses mediate this range of effects on biological processes using so few proteins. These include direct interactions with plant transcription and translation machinery that have been shown to induce extensive changes in the transcriptome [18,19,20,21]. Not surprisingly, many of the genes that are differentially regulated in response to begomovirus infection are involved in plant defense, including components of the transcriptional and post-transcriptional gene silencing pathways (TGS and PTGS, respectively), as well as jasmonic acid (JA) and salicylic acid (SA) signaling responses, and autophagy.

While virus–plant interactions have been explored at the transcriptional level for begomoviruses such as tomato yellow leaf curl virus (TYLCV) and tomato yellow mosaic virus (ToYMV), few global protein-level analyses have been performed. In addition, the begomovirus–host plant pathosystem also includes an insect vector, which in this case is the whitefly *Bemisia tabaci*. Several studies have examined the proteome of *B. tabaci* either in the presence [22] or absence [23,24] of begomoviruses. Furthermore, avirulent *B. tabaci* was recently found to impact redox regulation, stress response, as well as lipid and carbon metabolism in susceptible pepper genotypes 2 days post feeding (dpf) [25]. However, to the best of our knowledge, no study to date has examined the impact of whitefly-feeding and whitefly-mediated transmission of a begomovirus on a plant host. Therefore, we used the *B. tabaci*—tomato (*Solanum lycopersicum*)—tomato mottle virus (ToMoV) pathosystem to expand our understanding of the interactions between insect, pathogen, and host.

ToMoV was first described in 1992 [26,27], and it is a whitefly-transmitted ssDNA bipartite begomovirus that primarily infects solanaceous crop plants such as tomato. Unlike commercial tomato cultivars, the cultivar “Florida Lanai”, with its small size, rapid growth, and ease of maintenance has been shown to be an excellent model host for studying begomovirus pathogenesis [28]. It was observed that ToMoV accumulation in planta in ‘Florida Lanai’ is detectable as early as 7 days post infection (dpi) and exhibits symptoms as early as 7–10 dpi including stunted growth, mottled leaves, and eventually reduced fruit yield. In addition, ‘Florida Lanai’ has been previously used to assess the impact of ToMoV on various aspects of the whitefly life cycle [29]. Thus, the ToMoV and ‘Florida Lanai’ system is an effective model system to investigate differences in the plant proteome during whitefly and ToMoV pathogenesis.

In this report, we characterize and compare protein profiles of tomato leaves that were exposed to feeding by either non-viruliferous whiteflies (+WF), or viruliferous whiteflies carrying ToMoV (+WF^V^). Tomato leaves were sampled from two locations, including locally from a mature leaf isolated at the site of whitefly feeding (SOF) and systemically from leaves distal to the SOF. In each case, these profiles were compared to the leaf proteome of a third set of “no-feeding control” (NFC) tomato plants not exposed to either whitefly feeding or ToMoV. Our findings provide evidence of a predominant whitefly-mediated response at the SOF, and they also identified biological processes altered by ToMoV early in pathogenesis at 3 dpf. In younger leaves at 10 dpf, our experiment found large-scale systemic changes in the tomato proteome and identified multiple biological processes affected specifically by ToMoV. Our study of the proteome in systemic leaves is consistent with other similar studies [30,31,32,33,34] and improves the body of knowledge on host responses to whitefly feeding and begomovirus infection occurring early during infection, prior to the appearance of symptoms in systemic tissue. Furthermore, our observations identified many novel plant biological processes that are altered specifically in response to ToMoV in systemic leaves and provides a more in-depth picture of protein-level responses during tomato–whitefly–ToMoV interactions.

## 2. Results

### 2.1. Obvious Plant Growth Defects Were Observed Only 10 Days Post Whitefly Feeding (dpf) in Tomato Subjected to Feeding by Viruliferous Whiteflies (+WF^V^)

**Figure 1:**
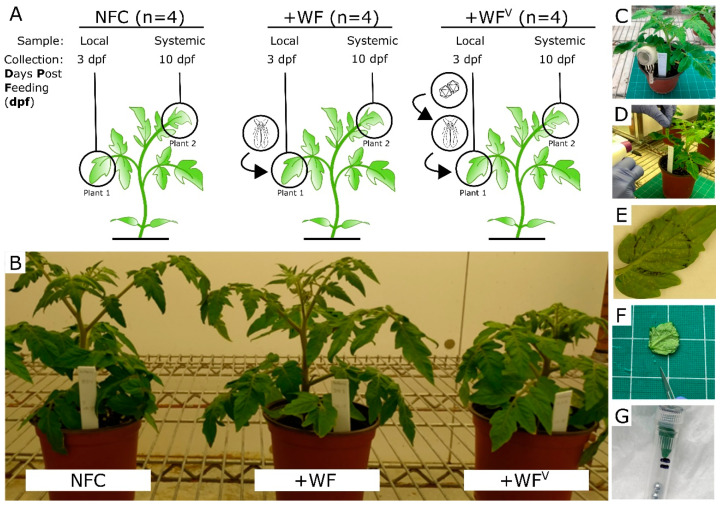
(**A**) The experimental design involved subjecting tomato plants to infection by whiteflies (+WF), viruliferous whiteflies loaded with tomato mottle virus (+WF^V^), and a no-feeding control (NFC). Leaf samples were collected from the whitefly site of feeding (SOF) at 3 days post feeding (dpf) and systemically from younger leaves at 10 dpf. Separate plants were used to sample leaves from the SOF at 3 dpf and leaves distal to the SOF at 10 dpf (plant 1/plant 2). (**B**) Representative plants at 10 dpf. Only the plants subjected to feeding by viruliferous whiteflies exhibited stunted growth and mottled leaves, which are characteristic of ToMoV infection. (**C**–**G**) All leaves, including the NFC, received clip cages. Additional sample handling included the removal of clip cages, whitefly termination by insecticidal soap, excision of the leaf, and isolation of the leaf area at the SOF within the clip cages.

As shown in Figure 1, we performed an experiment to determine how tomato leaves respond at the protein level to feeding by non-viruliferous whiteflies (+WF) and viruliferous whiteflies (+WF^V^) harboring ToMoV. Our experiment also included samples collected locally 3 days post feeding (dpf) directly from the whitefly site of feeding (SOF) and systemically at 10 dpf in newly formed leaves distal to the SOF. At both 3 and 10 dpf, protein abundances were also compared to samples from a no-feeding control (NFC) leaf that received neither +WF nor +WF^V^ (Figure 1A). In an attempt to maximize our ability to detect responses specific to ToMoV and minimize a large whitefly-mediated stress response, we confined 40 adult whiteflies to clip cages placed on mature leaves and limited the duration of feeding to 3 days, at which point the whiteflies were terminated (Figure 1C–G). The presence of ToMoV in all plants subject to +WF^V^ feeding was confirmed via nanopore sequencing. At 3 dpf, plants subjected to +WF, +WF^V^, and the NFC appeared indistinguishable. In contrast, at 10 dpf, the plants subjected to +WF^V^ feeding compared to the +WF and NFC plants exhibited mottled leaves and stunted growth characteristic of ToMoV infection (Figure 1B) [28]. These observations suggest that the 3-day period of +WF feeding in clip cages was insufficient to trigger the stunted growth phenotype observed in +WF^V^ plants at 10 dpf that is caused by ToMoV infection [28].

### 2.2. The Protein Profile of Tomato Leaves is Altered Predominantly by Whitefly Feeding at 3 dpf and Systemically by Both Whitefly and ToMoV at 10 dpf

**Figure 2:**
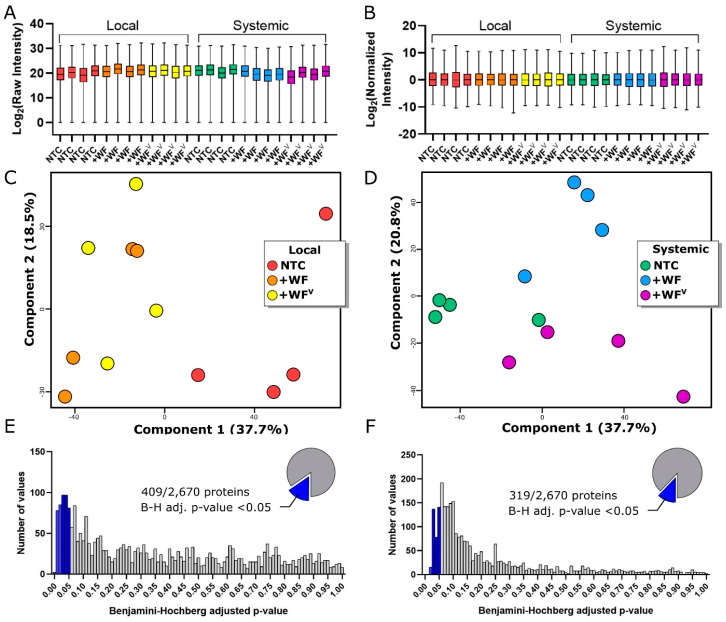
(**A**,**B**) Range of protein abundances pre-normalization (**A**) and post-normalization (**B**). (**C**,**D**) Principal component analysis for both local (**C**) and systemic (**D**) leaves collected 3 dpf and 10 dpf, respectively. Distribution of ANOVA *p*-values indicate the abundance of 409 and 319 proteins are significantly changed in local leaves at the SOF 3 dpf (**E**) and systemic leaves (**F**), respectively, after Benjamini–Hochberg multiple hypothesis test correction using a 5% false discovery rate (FDR).

Our LC-MS/MS proteomics analysis confidently identified 2670 proteins by considering only those proteins identified by ≥2 unique peptides, and occurrence in all four biological replicates of at least one treatment (e.g., all four +WF leaf samples at 3 dpf). The data were log_2_ transformed, median normalized, and analyzed first by principal component analysis (PCA) (Figure 2A–D). For leaves sampled from the SOF at 3 dpf, both PCA components 1 and 2 comprise 37.7% and 18.5% of data variation, respectively (Figure 2C). The predominant separation occurs between the NFC control samples and both +WF and +WF^V^ samples. The lack of separation on principal component 1 between the +WF and +WF^V^ samples at 3 dpf suggests that the acute changes in the leaf proteome at the SOF are dominated by the whiteflies’ presence. In contrast, the PCA of samples from systemic leaf at 10 dpf (Figure 2D) show the separation of +WF, +WF^V^, and the NTC, suggesting a distinct systemic impact of ToMoV on the proteome of younger tissue.

**Figure 3:**
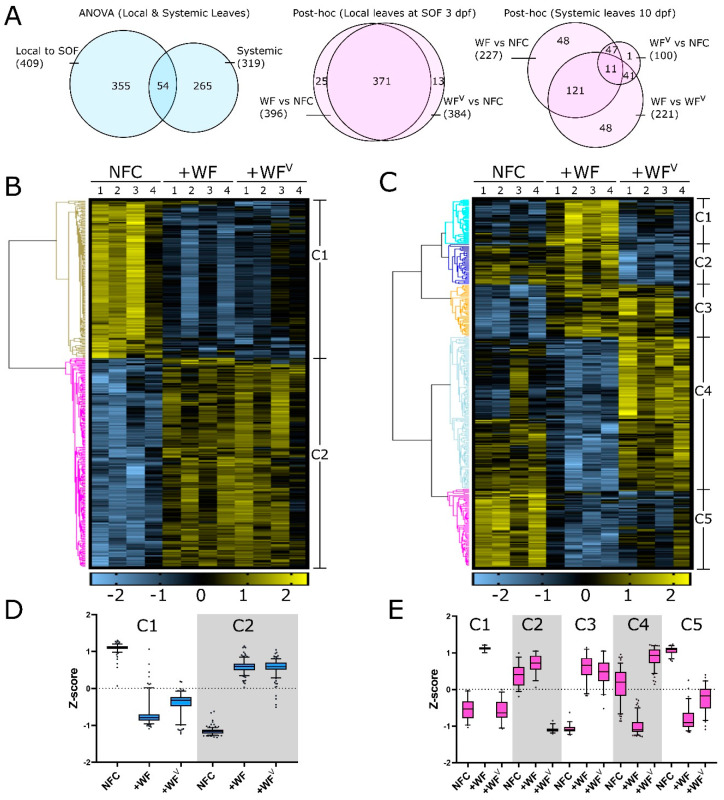
(**A**) Venn diagrams depicting number of proteins significantly changed in both local and systemic leaves by ANOVA (left, blue), as well as using pairwise post-hoc comparisons of treatments at the SOF 3 dpf (pink, middle) and in systemic leaves 10 dpf (blue, right). (**B**,**C**) Heat maps of hierarchical clustering analysis using z-score transformed data for local leaves 3 dpf (**B**) and systemic (**C**) leaves 10 dpf. (**D**,**E**) Cluster trends for both local (**D**) and systemic (**E**) leaves. Error bars represent the 5-95% z-score range for each sample. WF, whitefly; WF^V^, viruliferous whitefly; NFC, no-feeding control; SOF, Sight of feeding; C, Cluster.

Analysis of variance (ANOVA) performed using NTC, +WF, and +WF^V^ identified 409 and 319 proteins whose abundance significantly changed in samples from the SOF at 3 dpf and from systemic leaves at 10 dpf, respectively (B-H *q*-value < 0.05, Figure 2E,F). Only 54 proteins were found via ANOVA to be significantly changed in both samples from the SOF 3 dpf and systemic tissue at 10 dpf, suggesting that responses to pathogenesis manifested in the proteome changes over time, or as a function of leaf age (Figure 3A). To better understand the regulation of these proteins, we performed hierarchical clustering analysis (HCA), which resulted in the identification of two and five clusters for SOF 3 dpf and systemic leaf samples 10 dpf, respectively (Figure 3B,C). Within leaves at the SOF, the HCA cluster C1 (*n* = 176) contains proteins that apparently decrease in abundance in both +WF and +WF^V^ compared to the NTC, while the opposite trend is observed in cluster C2 (*n* = 233) (Figure 3B,D). A post-hoc pairwise analysis of +WF and +WF^V^ samples from leaves at the SOF 3 dpf revealed that only 13/409 proteins were significantly changed exclusively upon feeding by +WF^V^ (Figure 3A, middle). Furthermore, no significant changes were observed at the SOF 3 dpf in a post-hoc pairwise comparison of +WF and +WF^V^ protein abundances (B-H adj. *q*-value < 0.05). These data suggest that the protein profiles of leaves at the SOF at 3 dpf are altered predominantly by whitefly feeding, and the impact of ToMoV on tomato leaves at the SOF is relatively small or not well represented in our data. However, we identified 13 proteins that changed significantly using pairwise post-hoc analysis only between +WF^V^ and NTC, and not +WF versus NTC, suggesting the presence of a relatively small response to ToMoV early in viral pathogenesis.

In contrast to leaf samples collected at the SOF 3 dpf, the HCA generated from protein profiles of systemic leaves 10 dpf resulted in five clusters (Figure 3C,E). Clusters C1 (*n* = 39) and C4 (*n* = 133) consist of proteins whose abundance increases or decreases, respectively, in response to +WF feeding compared to the NFC. Similarly, proteins in clusters C4 and C2 (*n* = 35) tend to be more or less abundant, respectively, in response to feeding by +WF^V^ compared to the NFC. Clusters C3 (*n* = 45) and C5 (*n* = 67) contain proteins that are changed similarly by both +WF and +WF^V^ feeding with respect to the NTC. Interestingly, HCA clusters C1 and C2 contain proteins that are much less abundant in +WF^V^ samples as compared to +WF. Comparably, proteins in C4 (and to a lesser extent C5) are more abundant in +WF^V^ samples compared to +WF. Indeed, a pairwise post-hoc analysis indicates that the abundances of 221/309 tested proteins were significantly changed when comparing systemic +WF and +WF^V^ samples 10 dpf (Figure 3A, right) (B-H adj. *q*-value < 0.05). Assessed in aggregate, these data suggest that changes in the leaf proteome at the SOF is primarily a consequence of WF feeding at 3 dpf, while an additional ToMoV response is apparent in systemic tissues at 10 dpf. Moreover, these data also suggest that in some cases, ToMoV infection and the resulting pathogenesis involves the partial reversal of changes in protein abundance induced systemically by WF feeding (e.g., C1, C2, and C4).

### 2.3. Specific Biological Processes Are Altered by Whitefly Feeding and ToMoV Infection Both at the SOF 3 dpf and Systemically in Leaves 10 dpf

To evaluate the biological processes affected by feeding of +WF and +WF^V^ in leaves at the SOF 3 dpf and in systemic leaves 10 dpf, we performed gene ontology (GO) enrichment analyses using the proteins from each HCA cluster. Multiple GO slim terms were significantly enriched among proteins in each cluster, some of which are highlighted in Table 1 (Bonferroni adj. Fisher’s Exact *p*-value < 0.05, PANTHER) [35]. For example, in leaves at the SOF, proteins involved in translational elongation (GO:0006414) were significantly enriched approximately three-fold greater than expected by chance in clusters C1 and C2. These data suggest that aspects of mRNA splicing and translation are altered at the SOF 3 dpf. In systemic leaves at 10 dpf, we observed a significant 36-fold enrichment for two proteins in HCA cluster C1 associated with chromatin silencing (GO:0006342). Similarly, HCA cluster C2 in systemic leaves, which became less abundant in the presence of the virus, was enriched 3.5-fold for five proteins associated with RNA metabolism (GO:0016070). Clusters C1 and C2 in systemic leaves at 10 dpf contain proteins that are less abundant in leaves subject to +WF^V^ feeding as compared to non-viruliferous +WF leaves, suggesting that specific RNA processes are affected negatively by ToMoV infection. Conversely, proteins in systemic HCA cluster C4 at 10 dpf were positively affected specifically during ToMoV infection at 10 dpf and are significantly enriched with proteins associated with DNA demethylation, oxidative stress, and DNA repair. The comprehensive list of each GO term and their contributing proteins can be found in Supplemental Table S1.

### 2.4. Proteins Involved in Alternative Splicing, Translation, and Plasmodesmata Dynamics Are Affected 3 dpf by +WF and +WF^V^ in Leaves at the SOF

Enrichment analyses suggested that RNA metabolism is altered in leaves upon feeding by both +WF and +WF^V^. We observed thirty proteins associated with nucleic acid binding (GO:0003676) or RNA binding (GO:0003723) in HCA clusters C1 and C2 at the SOF. Ten of the 30 likely RNA-binding proteins cluster in HCA C1 at the SOF and include DNA-directed RNA polymerase (Solyc05g053430), as well as three CCHC-type zinc-finger domain proteins (Solyc03g033500, Solyc10g055250, and Solyc01g111275), each of which were significantly 5.9−, 5−, 4.9−, and 4-fold less abundant, respectively, in +WF samples as compared to NFC (B-H *q*-values < 0.05). Interestingly, while these proteins were also significantly less abundant in +WF^V^ samples as compared to NFC, the magnitude of their decrease was attenuated, with only a 3.2−, 3−, 2.4−, and 2.3− fold decrease, respectively. Furthermore, in systemic leaves at 10 dpf, each of these proteins clustered in HCA cluster C4, and they were found to be significantly decreased in response to +WF feeding but significantly increased upon feeding by +WF^V^ as compared to the NFC. Therefore, overlapping members of cluster C1 from the SOF at 3 dpf and cluster C4 from systemic leaves at 10 dpf may constitute some of the earliest changes in tomato leaf protein profiles in response to ToMoV infection. Most of the proteins that changed in leaves at the SOF 3 dpf were altered similarly upon feeding by both +WF and +WF^V^ (Figure 3A, middle). Among the 13 proteins altered upon feeding by +WF^V^ but not +WF as compared to the NFC are the eukaryotic translation initiation factors (eIF) (Solyc12g009960: eIF4G domain-containing and Solyc10g079880: eIF3e), both of which were significantly decreased by 3.5− and 2.5−fold only upon +WF^V^ feeding (B-H *q*-value < 0.05). Orthologs of these two eIF proteins from Arabidopsis are known to be involved in plant viral immunity [36], and these data suggest that Solyc12g009960 and Solyc10g079880 may similarly be part of the early response to ToMoV infection in tomato.

The remaining 20 likely RNA-binding proteins in leaves clustered in C2 at the SOF 3 dpf, and they include two SM-domain containing proteins associated with mRNA splicing (Solyc10g078450 and Solyc09g072970), each of which were significantly elevated approximately 2.5-fold in response to both +WF and +WF^V^ feeding as compared to NFC (B-H *q*-values < 0.05). These data suggest that aspects of mRNA splicing are altered by both +WF and +WF^V^ feeding at 3 dpf. Additional RNA-binding proteins in HCA cluster C2 at the SOF include two RBP45-like polyadenylate binding proteins (PABPs), Solyc10g005260 and Solyc12g088720, which likely function in RNA stability and stress adaptation [37,38]. The PABP (Solyc12g088720) is significantly approximately 2.1-fold more abundant in both +WF and +WF^V^ samples as compared to the NFC. Interestingly, the RBP45b-like PABP is significantly 3.5-fold more abundant in +WF^V^ samples as compared to NFC, but not in +WF samples. Moreover, the abundance of Solyc10g005260 is also significantly increased in the systemic leaves of plants subject to +WF^V^ feeding at 10 dpf, clustering in HCA C4. The closest Arabidopsis ortholog of Solyc10g005260 is the cell-to-cell mobile RNA binding protein AtRBP45b (At1g11650, BLASTp results found in Supplemental Table S1). Taken together, these data suggest that distinct populations of RNA-binding proteins associated with mRNA splicing and stability respond early to +WF and +WF^V^ at the SOF at 3 dpf, and that the RBP45b-like PABP response may be ToMoV-specific.

In addition to RNA metabolism, we observed a change in three proteins involved in callose deposition at the plasmodesmata neck mediated by both +WF and +WF^V^ feeding. For example, the X8-domain containing proteins Solyc07g047710, Solyc03g115220, and Solyc07g053430 are significantly less abundant in +WF as compared to NFC, with decreases of 54-, 7-, and 5-fold respectively, and they exhibit similar decreases in +WF^V^ samples (B-H *q*-value < 0.05). Similarly, the likely sieve element occlusion protein Solyc03g111820 was 51-fold less abundant in leaves from plants subject to +WF and +WF^V^ feeding as compared to the NFC (B-H adj. *q*-value < 0.01). These data suggest that callose deposition at the plasmodesmata and sieve element occlusion may be reduced, which may facilitate phloem cell-to-cell conductance early in pathogenesis by both WF and +WF^V^.

### 2.5. Tomato Chromatin Architecture and Alternative Splicing Are Affected by ToMoV in Systemic Leaves 10 dpf

**Figure 4:**
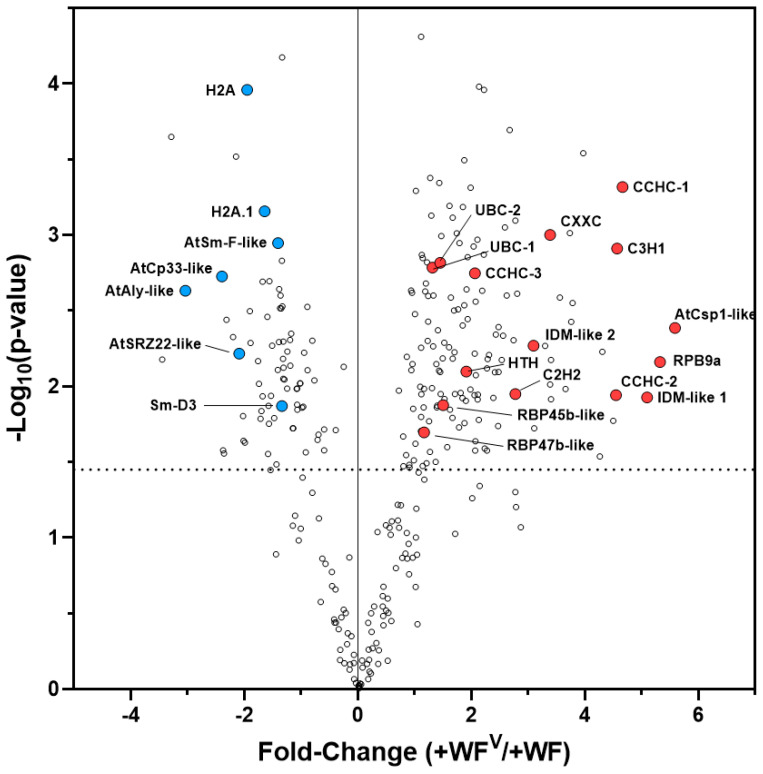
Volcano plot of pairwise +WF and +WF^V^ comparison from systemic leaves at 10 dpf identified multiple proteins whose abundance changed significantly as a specific response to tomato mottle virus (ToMoV) (B-H *q*-value < 0.05). AtCsp1-like, Solyc03g033500; RBP9a, Solyc05g053430; IDM-like 1, Solyc01g098810; CCHC-1, Solyc02g083800; C3H1, Solyc11g008900; CCHC-2, Solyc10g055250; CXXC, Solyc11g070050; IDM-like 2, Solyc04g082720; C2H2, Solyc09g013120; CCH3, Solyc01g111275; RBP45-like, Solyc10g005260; UBC-1, Solyc01g007860; UBC-2, Solyc10g083120; RPB47b-like, Solyc10g050860; Sm-D3, Solyc03g033600; Sm-F-like, Solyc10g081370; H2A.1, Solyc01g099410; H2A, Solyc11g073250; AtSRZ22-like, Solyc08g069120; AtCp33-like, Solyc01g006940, AtAly1-like, Solyc11g073280. Individual abundance values, fold-changes, and *p*-values can be found in Supplemental Table S1.

The abundance of proteins identified at 10 dpf within systemic leaf HCA clusters C1 and C2 are largely reduced specifically by ToMoV or by the host tomato in response to ToMoV relative to +WF-treated plants (Figure 3E). Among these C1 and C2 clustered proteins, our GO enrichment analysis indicates that the ontologies chromatin silencing (GO:0006342) and RNA metabolism (GO:0016070) are significantly 36- and 3.5-fold enriched, respectively (Table 1). These data suggest that RNA processes are altered specifically during ToMoV pathogenesis. Members of cluster C1 associated with the chromatin silencing ontology include histone 2A (H2A, Solyc11g073250) and the variant histone 2A.1 (H2A.1, Solyc01g099410), both of which were significantly more abundant (approximately 2-fold) in systemic +WF leaf samples, but then became significantly approximately 3-fold less abundant in +WF^V^ leaf samples as compared to +WF (Figure 4). Both H2A and H2A.1 are likely involved in chromatin structure and DNA accessibility [39]. Protein abundance of the RRM-domain containing protein Solyc11g073280 was significantly 8.2-fold reduced in systemic +WF^V^ leaf samples as compared with systemic +WF leaf samples at 10 dpf (B-H *q*-value < 0.05). The Arabidopsis ortholog with the greatest homology to Solyc11g073280 is At5g59950 (AtALY1, BLASTp results found in Supplemental Table S1), which was recently shown to facilitate global RNA-directed DNA methylation (RdDM) and subsequent transcriptional gene silencing (TGS) [40]. The observation that proteins associated with RNA-directed gene silencing and chromatin accessibility appear to become more abundant in response to feeding by +WF but decrease to levels comparable to the NFC specifically in response to feeding by +WF^V^ suggests that ToMoV pathogenesis includes suppression of the host gene silencing pathway that is apparently triggered systemically by whitefly feeding. This is consistent with reports that begomoviruses suppress TGS via the action of viral pathogenicity factors [32,33].

Similarly, proteins in HCA cluster C2 were less abundant in +WF^V^ samples as compared to both +WF and NFC samples. Enriched among these C2 proteins is the ontology RNA metabolism (GO:0016070), which includes the CCHC-domain containing protein Solyc08g069120 as well as the Sm-domain containing proteins Solyc03g033600 (Sm-D3) and Solyc10g081370 (Sm-F like), each of which were significantly reduced 4.2-, 2.5-, and 2.6-fold, respectively, in +WF^V^ samples as compared to +WF (B-H *q*-value < 0.05) (Figure 4). The CCHC-domain containing protein is homologous to the Arabidopsis mRNA splice factor and nuclear exporter AtSRZ22 (At4g31580, BlastP results in Supplemental Table S1) [41], while Solyc10g081370 (nearest Arabidopsis ortholog At4g30220 Sm-F, Supplemental Table S1) and Solyc03g033600 (Sm-D3) are predicted to encode small nuclear ribonucleoproteins involved in pre-mRNA splicing [42,43]. We also observed a significant 5.3-fold decrease in an RRM domain containing protein (Solyc01g006940) in systemic leaves upon feeding by +WF^V^ as compared to feeding by +WF. The closest Arabidopsis ortholog to Solyc01g006940 is AtCp33 (At3g52380, Supplemental Table S1), which was shown to stabilize and prevent the degradation of ribosome-free chloroplast mRNA [44,45]. The observation that these proteins become less abundant specifically in +WF^V^ samples, but not to +WF feeding alone, suggests a ToMoV specific modulation of mRNA splicing and degradation processes.

### 2.6. RNA-Directed DNA Methylation and Other RNA Maturation Processes Are Altered in Systemic Leaves during ToMoV Pathogenesis

In systemic leaves at 10 dpf, the abundance of proteins in HCA cluster C4 are increased specifically in response to feeding by +WF^V^ compared to both non-viruliferous +WF and NTC samples (Figure 3E). Among these cluster C4 proteins, we observed a significant 4-fold enrichment for members of the DNA-repair ontology (GO:0006281) (Table 1). One member of this ontology, Solyc05g053430, was significantly 40-fold more abundant in +WF^V^ systemic leaf samples as compared to +WF samples (B-H *q*-value < 0.05). The closest Arabidopsis ortholog of Solyc05g053430 is AtRPBa (At3g16980, Supplemental Table S1), which may be involved in RNA-dependent DNA methylation (RdDM) and gene silencing [46]. The remaining two members of the DNA-repair ontology are Solyc01g007860 and Solyc10g083120 (annotated here as UBC-1 and UBC-2, respectively), both of which are predicted E2-ubiquitin conjugating (UBC) enzymes, the closest Arabidopsis ortholog of which is AtUEV1D (At3G52560, Supplemental Table S1). Both UBC-1 and UBC-2 are significantly 2.5-fold more abundant in +WF^V^ systemic leaf samples compared with the +WF group (Figure 4). Previous studies have shown AtUEV1D is involved in transcription across lesions in damaged DNA [47]. Proteins in the demethylation ontology (GO:0070988) were significantly 40-fold over-represented among cluster C4 proteins. This includes Solyc01g098810 and Solyc04g082720, which are 32- and 8-fold more abundant, respectively, in systemic +WF^V^ leaf samples as compared to +WF samples 10 dpf (B-H *q*-value < 0.05). These proteins are related to IDM2/3-like small heat shock proteins (sHSPs) whose closest Arabidopsis orthologs have been shown to be involved in DNA demethylation [48,49]. Interestingly, both IDM2/3-like sHSPs were significantly less abundant in leaf samples from the SOF at 3 dpf (local HCA cluster C1) but became significantly more abundant in systemic +WF^V^ leaves 10 dpf (systemic HCA cluster C4). Together, these data are consistent with previous reports that increased DNA methylation is an active plant defense response against begomovirus infection [31]. Our observation that at 10 dpf, the IDM2/3-like DNA demethylation proteins become more abundant in samples from the +WF^V^ group, but not the +WF group, suggests a systemic ToMoV-specific anti-RdDM counter defense, which is likely to increase the demethylation of the viral genome and suppress plant TGS.

In systemic leaves at 10 dpf, the proteins exhibiting the largest significant increase in abundance of between 2- and 50-fold specifically in +WF^V^ systemic leaf samples as compared to +WF are zinc-finger domain proteins including Solyc03g033500 (CCHC), Solyc02g083800 (CCHC), Solyc10g055250 (CCHC), Solyc11g008900 (C3H1), Solyc11g070050 (CXXC), and Solyc09g013120 (CCHH) (B-H *q*-value < 0.05, Figure 4, Supplemental Table S1). The closest Arabidopsis orthologs of tomato CCHC, C3H1, and CCHC proteins are cold-shock proteins that may have roles in modulating the mRNA secondary structure and translation efficiency (Blastp results in Supplemental Table S1). Interestingly, the tomato CCHH (Solyc09g013120) ortholog in Arabidopsis is At5G01160, which was recently shown to be involved in mRNA methylation [50]. We also observed a significant ToMoV-specific increase in the abundance of two likely polyA–mRNA binding proteins, Solyc10g005260 (3.7-fold) and Solyc10g050860 (3.2-fold) (B-H *q*-value < 0.05). The Solyc10g050860 protein is homologous to Arabidopsis RBP47b (At3g19130, Supplemental Table S1), which is known to play a role in stress granule formation [51,52] caused by translational repression and stalled translation initiation [53,54,55]. Similarly, Solyc10g005260 is homologous to Arabidopsis RBP45b (At1g11650, Supplemental Table S1), which has also been shown to accumulate in stress granules. These data suggest that RNA-binding proteins involved in translation efficiency are increased during ToMoV infection, but not by +WF feeding alone. This is also consistent with previous reports that demonstrate that the SnRK1 phosphorylation of eIF4E and eIFiso4E inhibits translation [34], and that begomoviruses inhibit SnRK1 activity to suppress SnRK1-mediated defense responses [30].

### 2.7. Similar DNA Motifs Are Found Upstream of Genes Regulated by ToMoV in Systemic Leaves at 10 dpf

We next determined whether specific DNA sequence motifs were common amongst the promoter regions for genes encoding proteins regulated specifically by ToMoV in systemic leaves at 10 dpf. The 2000 base pairs upstream of the coding region for the top 20 genes corresponding to proteins with the greatest increased or decreased abundance changes in systemic leaves at 10 dpf were analyzed. A total of two and three common motifs were revealed upstream of upregulated and downregulated genes, respectively via MEME-suite (*p*-value < 0.05). Summarized in Table 2, all but one motif was identified to be significantly enriched upstream of Arabidopsis genes associated with transcription factor activity (*q*-value < 0.05). Interestingly, the second motif identified upstream of gene products increased specifically by ToMoV was also found upstream of genes associated with ubiquitin ligase activity, the production of long small interfering RNA (lsiRNA), leaf development, and circadian rhythm. This suggests that ToMoV infection induces components of these cellular pathways possibly through the action of common transcriptional regulator(s). Similarly, three motifs identified among the 20 promoters of genes encoding proteins that decreased in response to ToMoV are also enriched upstream of Arabidopsis genes associated with leaf development, response to water deprivation, and response to jasmonic acid stimulus. Thus, ToMoV infection appears to suppress components of these pathways, again potentially through a transcriptional regulator(s). Further experiments are necessary to determine if these sequences are involved in regulating tomato transcript-level response to ToMoV. The consensus sequences for each motif identified by the MEME search, and other ontologies found to be enriched by GOMo can be found in Supplemental Table S1.

## 3. Discussion

Begomoviruses are ssDNA plant viruses transmitted by whiteflies that cause significant diseases in crop plants worldwide. Proteomic analyses have been performed previously to identify interactions occurring between the whitefly and begomoviruses [22], and to identify proteins differentially expressed in response to over-expression of the begomovirus AC2 transactivating protein [56]. However, to the best of our knowledge, this work is the first comprehensive proteomic examination of a host plant response to feeding by non-viruliferous whiteflies (+WF) and viruliferous ToMoV-loaded whiteflies (+WF^V^) in both mature leaves at the site of feeding (SOF) and in newly formed systemic leaves developed after confined whitefly feeding 10 days later. To focus our experiment on the tomato response to ToMoV, we sought to reduce any potential large-scale plant stress response induced by whitefly feeding by limiting the extent and duration of their feeding. At the time of whitefly removal 3 days post feeding (dpf), both +WF and +WF^V^ plants appeared indistinguishable from the no-feeding control (NFC) plants, which is consistent with an absence of WF-mediated growth retardation under our experimental settings. Conversely, at 10 dpf, plants subjected to feeding by viruliferous whiteflies (+WF^V^) exhibited stunted growth and mottled newly formed leaves, which is characteristic of a systemic ToMoV infection.

Our analysis resulted in the confident identification and quantification of 2670 tomato proteins. Follow-up analysis of variance (ANOVA) indicates that the abundance of 409 and 319 proteins were significantly altered in leaves 3 dpf at the site of feeding (SOF) and systemically 10 dpf, respectively. At the SOF, principal component analysis (PCA) and hierarchical clustering analysis (HCA) indicated that host plants at 3 dpf responded similarly to feeding by both +WF and +WF^V^, suggesting a relatively small additional effect early in ToMoV pathogenesis. This is consistent with previous reports that whiteflies can suppress a host response at 3 days post infection [57], and a lack of observable virus impact until 4 dpf [28]. Unlike leaves 3 dpf at the SOF, our PCA and HCA suggests that the protein profiles of systemic leaves at 10 dpf appear to be impacted by the presence of the virus in addition to whitefly feeding, with proteins in clusters C1, C2, and C4 appearing to respond specifically to ToMoV. Interestingly, the trends in protein abundance for systemic leaf clusters C1 and C4 appear to be opposite for +WF and +WF^V^ plants. Together, this suggests that in some cases, there may be a ToMoV-mediated suppression of a systemic host response to WF feeding.

Of the differentially expressed proteins identified 3 dpf at the SOF, several are of potential relevance for begomovirus pathogenicity. The significant decrease in levels of X8-domain containing proteins (Solyc07g053430, Solyc07g047710, Solyc03g115220) and a sieve element occlusion-domain containing protein (Solyc03g111820) by both +WF and +WF^V^ may have implications for viral movement. Some X8-domain proteins have been found to localize to the plasmodesmata, bind β-1,3-glucans, and when overexpressed *in-planta* lead to increased callose deposition, decreased plasmodesmata size-exclusion limits, and decreased cell-to-cell mobility [58]. Similarly, sieve element occlusion protein family members are thought to be involved in limiting translocation across the phloem sieve [59], but their exact role is debatable in the literature [60]. Although the function of these proteins in facilitating and/or inhibiting viral pathogenesis is unclear, there are previous data that provide insight into a possible role. All bipartite begomoviruses encode a nuclear shuttle protein (NSP) and a movement protein (MP) that are required for cell-to-cell and long-distance movement of the virus. Earlier studies have shown that the *Bean dwarf* mosaic virus MP alters the size exclusion limit of plasmodesmata to promote movement between cells [61], and that the MP encoded by *Squash leaf curl* virus induces the formation of ER-derived tubules to allow the transport of a viral protein–DNA complex to adjacent cells [62]. The observation that the abundance of these proteins was similarly changed in both +WF and +WF^V^ compared to the NTC suggests that this is a whitefly-mediated response. It is interesting to speculate that a reduction in the level of proteins associated with limiting cell-to-cell mobility and phloem transport in local tissues upon feeding by *B. tabaci* may facilitate begomovirus movement early in pathogenesis, thereby allowing the virus to move systemically before being detected by the plant. It should be noted that in systemic leaf tissue, two additional X8 domain containing proteins (Solyc12g019890 and Solyc08g005000) are more abundant after both +WF and +WF^V^ feeding, while another (Solyc09g057630) is reduced during +WF^V^ compared to +WF and NTC. While the reason for this is not clear, it is possible that there are different requirements for virus movement in younger tissue.

Interestingly, at the SOF 3 dpf, we did identify 13 proteins that responded significantly only to +WF^V^ compared to the NFC, but not in +WF alone. For example, the likely eukaryotic initiation factor (eIF) 4G-domain containing protein (Solyc12g009960) and eIF3 protein (Solyc10g079880) were significantly reduced only in +WF^V^ leaves compared with the NTC. The closest orthologs of the tomato eIF4G-like protein are the Arabidopsis eIFiso4G protein (AT5G57160) and Rice eIFiso4G (Os04g0499300, Blastp results in Supplemental Table S1). Translation of mRNAs in eukaryotes is mainly cap-dependent, and different initiation factors are involved in the assembly of an mRNA–protein complex [63,64]. The initiation of translation involves the assembly of an eIF4F protein complex consisting of a cap-binding protein eIF4E, as well as eIF4G and eIF4A, which together binds polyadenylate-binding protein (PABP) and eIF3 [36,65]. Host eIF4G and eIFiso4G isoforms are commonly usurped by viruses [36], and it has been shown that the viral genome-linked protein (VPg) of rice yellow mottle virus (RYMV) interacts directly with eIFiso4G [66]. In plants, the eIF3 protein plays multiple roles in translation initiation [67], and it was also shown to enhance virus-activated translation re-initiation [68]. This is relevant because, similar to many plant viruses, begomoviruses do not encode translation factors, and therefore, the expression of viral genes necessitates the subversion of host translation factors, such as eIF4G, eIF3, eEF1, and likely others. Thus, downregulation of the two tomato eIF proteins could be an early plant antiviral defense mechanism to reduce translation of the viral mRNA. The evolution of translational suppression as a general plant defense against viruses, including begomoviruses, would provide a significant means to inhibit pathogenesis. Further, a number of host plant recessive resistance genes map to mutations in eIFs belonging to the eIF4E and eIF4G family or their isoforms eIFiso4E and eIFiso4G [69,70,71]. As an example, mutations in the rice eIFiso4G were shown to result in resistance to *Rice yellow mottle virus* (RYMV) [72]. Directly relevant to begomoviruses is a recent study showing that AtSnRK1, a homologue of mammalian AMP-activated kinase and yeast sucrose non-fermenting 1 (SNF1), inhibits translation by phosphorylating eIF4E and eIFiso4E [34]. This suggests a mechanism for the observation that SnRK1 facilitates an effective innate defense against begomoviruses [30].

The control of translation through the phosphorylation of eIF4E and eIFiso4E by SnRK1 represents a plant antiviral strategy that would provide a mechanism to prevent the translation of viral RNAs. This is supported by the demonstration that the begomovirus and curtovirus pathogenicity factors AL2 and L2 inhibit SnRK1 and plants expressing AL2 or L2 are more susceptible to infection [30]. Interestingly, the ToMoV specific changes in abundance of eIF4G-like (Solyc12g009960) and eIF3 (Solyc10g079880) were observed only at the SOF 3 dpf, suggesting they may constitute part of an early response to potentially viruliferous whitefly feeding that is later circumvented by viral counter defense. In contrast, additional translation initiation proteins including eIF5A-4, eIF1-like, and eIF5A-3 (Solyc12g010060, Solyc07g064150, and Solyc01g011000, respectively) were all significantly more abundant in both +WF and +WF^V^ groups at the SOF, but not in systemic leaves 10 dpf. Again, the specific role, if any, of these proteins in begomovirus pathogenesis is unknown. However, it may be possible that specific translation systems are affected by whitefly feeding, which may represent an initial defense response to a potentially viruliferous whitefly feeding.

**Figure 5:**
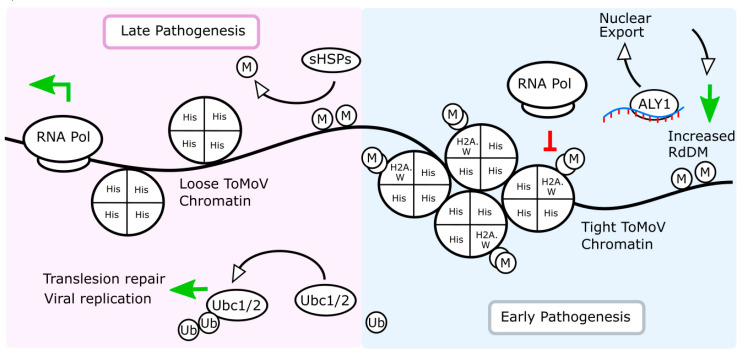
Interpretive model of ToMoV-specific protein changes identified in this study in transitioning from local early to late pathogenesis in systemic leaves. The increased abundance of ALY1-like protein (Solyc11g073280) early in pathogenesis at 3 dpf is consistent with an increased RNA-dependent DNA methylation (RdDM, green arrow, right) plant strategy to facilitate transcriptional gene silencing (TGS). Significantly less ALY1-like protein was observed in +WF^V^ at 10 dpf. Similarly, the likely- histone variants demethylated H2A and H2A.W (Solyc01g099410 and Solyc11g073250) which have been shown to associate with condensed and transcriptionally inactive heterochromatin (red “T”, right) became less abundant at 10 dpf in +WF^V^ leaves, suggesting a potentially relaxed heterochromatin state that may facilitate increased ToMoV gene expression. Other methylation states not shown in this model can also affect chromatin structure. An increase in UBC1 and 2 (Solyc01g007860 and Solyc10g083120), which have been associated with translesion repair, as well as the IDM-like sHSPs (Solyc01g098810 and Solyc04g082720) previously shown to decreased DNA methylation and relieve TGS (green arrows, left), were both more abundant late in pathogenesis at 10 dpf in response to ToMoV. His, non-specific histone; M, methyl group; RNA Pol, RNA-dependent DNA polymerase; sHSPs, IDM-like small heat shock proteins; Ubc1/2-like, ubiquitin conjugating protein; ALY1, Ally of AML-1 and LEF-1-like protein; ToMoV, tomato mottle virus; RdDM, RNA-dependent DNA methylation.

In contrast to the proteome in leaves sampled at 3 dpf, our results suggest that the leaf proteome is significantly altered specifically by ToMoV infection in systemic leaves at 10 dpf, and it includes the biological processes of RNA metabolism, TGS, post-TGS, and chromatin accessibility. This observation is relevant to begomovirus pathogenesis, given that they encode proteins that function to suppress TGS. As an example, we observed two histone proteins, H2A.1 and H2A (Solyc01g099410 and Solyc11g073250, respectively), that became more abundant systemically in response to +WF feeding but are significantly reduced in response to +WF^V^ feeding. While tomato H2A.1 returned to NFC levels in samples from the systemic +WF^V^ group, H2A abundance was further reduced when compared to the NFC. Homology analysis indicates that tomato H2A.1 and H2A are most closely related to the Arabidopsis H2A.W histone family members AtH2A.W.7 and AtH2A.W.12, respectively. In Arabidopsis, H2A.W histone variants associate with di-methylated histone H3 lysine 9 (H3K9me2) heterochromatin, which along with H3K27 methylation is one of the hallmarks of repressed chromatin and TGS [39,73,74]. This molecular interaction promotes chromatin condensation, which together with DNA methylation is known to cooperatively silence transposable elements [39,75]. Therefore, the apparent decrease in these histone variants, specifically in response to ToMoV, may constitute a viral countermeasure to plant TGS triggered by whitefly feeding (Figure 5).

Plants also employ RNA-dependent DNA methylation (RdDM) as an antiviral defense against DNA viruses to target and methylate viral genomes leading to suppressed gene expression and replication [17,57,76]. In systemic leaves of plants subject to +WF^V^ feeding, we observed a significant reduction of an RNA recognition motif (RRM) domain containing protein (Solyc11g073280). Sequence homology suggests that Solyc11g073280 is an ortholog of the AtAly1 protein in Arabidopsis and *Nicotiana benthamiana*. The Aly proteins are RNA-binding proteins required for mRNA nuclear export [77,78], and AtALY1 mutants were recently shown to globally reduce RdDM and suppress subsequent transgene silencing [40]. While AtAly1-mediated suppression of RdDM was also linked to decreased export of the transcript encoding ARGONAUTE6 [40], we observed no decrease in the ARGONAUTE proteins identified in this work. The P19 protein encoded by the tomato bushy stunt virus (TBSV) is a viral suppressor of host gene silencing, and it has been found to interact with multiple Arabidopsis ALY proteins, causing their redistribution in the cell [79,80]. The significant reduction in tomato AtAly-like protein during systemic ToMoV infection may also constitute a viral countermeasure to host plant RdDM antiviral defenses (Figure 5). Despite the trend of ToMoV-specific suppression of host RdDM observed in this experiment, we identified one protein, Solyc05g053430, that increased in response to the virus and may be involved in RdDM. Sequence homology suggests that Solyc05g053430 is most similar to Arabidopsis Rpb9a, a subunit of RNA Pol II, IV, and V. In addition to RNA Polymerases I–III, plant genomes also encode Pol IV and Pol V, which are known to mediate RdDM and suppress the transcription of transposons and viruses [81]. In Arabidopsis, Rpb9a and Rpb9b are two nearly identical but functionally non-redundant subunits of RNA Pol II, IV, and V [82]. Unlike most other RNA Pol subunits, Rpb9 is unique because it is not essential for mRNA synthesis, although it is necessary for growth under extreme temperatures [83]. Defects in RdDM were observed in Arabidopsis only in mutants of AtRpb9b but not AtRpb9a [46], but the specific role of these RNA Pol IV and V subunits remains to be evaluated in tomato.

We also observed increased levels of two “increased DNA methylation-like” (IDM) small heat shock proteins (sHSPs), Solyc01g098810 and Solyc04g082720, that were significantly more abundant in systemic leaves 10 dpf by +WF^V^ as compared to feeding by non-viruliferous +WF. Both of these tomato IDM-like sHSPs belong to the same α-crystallin domain containing family, and they are not induced immediately by heat, suggesting a function unrelated to protein folding [84]. Other α-crystallin sHSPs have been implicated in the pathogenesis of *Tobacco etch* virus in Arabidopsis [85]. The IDM2 protein in Arabidopsis interacts with IDM1, and IDM2 mutants are hypermethylated at thousands of loci, resulting in transcription silencing for multiple reporter genes, strongly suggesting that IDM2 is involved in DNA de-methylation [49]. Our observed increase in two IDM-like tomato proteins, specifically in response to systemic ToMoV infection, suggests that one aspect of the ToMoV-mediated counter defenses involves a global increase in DNA demethylation, which could lead to the suppression of plant TGS (Figure 5).

Overall, the observation that a significant number of proteins involved in TGS are differentially expressed in systemic tissue from plants infected with ToMoV is consistent with the role of TGS as a counter defense to plant antiviral strategies. A decrease in tomato H2A and H2A.1 may promote the relaxation of viral chromatin condensation, providing access for RNA pol II and a subsequent increase in viral transcription. Our data also demonstrate that ToMoV infection reduces the level of an AtALY-like protein abundance, which could lead to a decrease in RdDM-dependent viral gene silencing, as well as an increase in IDM-like sHSPs, which in turn would decrease viral DNA methylation and TGS. All of these changes are consistent with the ability of begomoviruses to counter plant antiviral defenses, including TGS [32,86]. Whether this is mediated through the action of the viral pathogenicity factors AL2/L2 is unknown; however, it would be an interesting line of investigation. Other proteins that could play a role during ToMoV infection are two ubiquitin conjugating domain-containing enzymes, Solyc01g007860 (UBC-1) and Solyc10g083120 (UBC-2), that were significantly more abundant in systemic leaves after feeding by +WF^V^ feeding compared to +WF at 10 dpf. Both of these proteins are homologous to Arabidopsis UEV1D, which is known to be involved in DNA damage repair [47]. While the immediate significance of this may not be apparent, the circular begomovirus ssDNA is converted to circular dsDNA by RNA-primed DNA polymerization [87], likely using translesion DNA polymerases [88]. Translesion polymerases help to repair DNA damage at stalled replication forks, which would have exposed regions of ssDNA. Thus, increased levels of proteins involved in DNA repair may afford begomoviruses the opportunity to convert their ssDNA genome into the dsDNA replicative intermediates that serve as templates for viral gene transcription and further rounds of rolling circle replication (Figure 5) [89].

Over the past few years, there has been an increase in potential links between nuclear RNA granules and other cellular RNA regulatory systems, including RNA silencing (for a review, see Makinen et al., 2017 [90]). It seems increasingly likely that the efficient viral infection of a host, both plants and mammals, requires components of stress granules (SGs) and processing bodies (PBs). Stress granules are nuclear structures that contain accumulated translationally inactive mRNAs during a stress response, including viral infection, and in some cases for regulating gene expression [91]. It is striking that some viral suppressors of RNA silencing localize to RNA granules, where they may function to suppress PTGS and/or TGS. Interestingly, two proteins, RBP45-like (Solyc10g005260) and RPB47b-like (Solyc10g050860), shown to accumulate in SGs [52], were significantly more abundant at 10 dpf in systemic leaves subjected to +WF^V^ feeding compared to the +WF alone, suggesting that their accumulation was ToMoV-specific. In Arabidopsis, AtRBP45a (At5g54900) binds to the primary driver of cell cycle progression CDKA;1 [92,93]. It has been shown that CDKA;1 and other plant innate immune-relevant [94] mitogen-activated protein (MAP) kinases (e.g., MKK5, MPK3, SNRK2.1 kinase) accumulate in SGs during stress, and it has been proposed that upon stress, AtRBP45b binds CDKA;1 and together with AtRBP47b are sequestered in stress granules, leading to arrest of the cell cycle [55]. Thus, these proteins are of potential significance with respect to plant antiviral mechanisms that suppress begomovirus infection for two reasons: (1) a potential role in suppressing the translation of viral mRNAs through the sequestration to SGs and (2) arrest of cell cycle, which would prevent the expression of plant genes necessary for viral DNA genome replication. Suppressors of RNA silencing encoded in the begomovirus genome could prevent the sequestration of viral mRNAs and/or inhibit SGs function as a viral counter-defense. It is also possible that this could prevent cell cycle arrest through prevention of the relocalization of CDKA;1. Additional support for a role of the begomovirus RNA silencing suppressors in maintaining the cell cycle in infected cells comes from observations that the inhibition of adenosine kinase (ADK) by the begomovirus AL2/L2 proteins results in the activation of primary cytokinin-responsive genes such as ARR5 [95]. This could potentially lead to an increase in the pool of bioactive cytokinin to promote cell proliferation, which is consistent with the demonstration that an exogenous application of cytokinin increases the kinetics of viral DNA accumulation [95].

Our study has identified hundreds of tomato proteins that differentially accumulate locally and systemically in response to feeding by whiteflies alone and/or whitefly-transmitted ToMoV. Many of these proteins appear to have orthologs that are involved in antiviral responses, and our findings are consistent with previous studies describing their role in antiviral defense. Whether these proteins are coordinately regulated as an antiviral response or are coordinately induced and/or repressed by geminivirus infection is currently unknown. However, analysis of the 2000 bp upstream of the coding region for the proteins most significantly altered specifically by ToMoV revealed multiple DNA motifs that were common to the promotors of those genes. The presence of putative Arabidopsis transcription factor binding motifs in promoter regions of tomato genes associated with plant–virus relevant biologically processes is suggestive of a common set of transcription factors that regulate genes involved in antiviral defense and/or a common set of genes that are targeted by geminiviruses during infection. However, the correlation between transcriptional control for these genes and protein abundance may be low as a result of PTGS, mRNA modification, and alternative splicing impacting the transcript stability or translation, as well as altered protein half-life caused by multiple mechanisms. Whether any of the proteins identified have direct or indirect roles in protecting the plant from begomovirus infection or conversely in promoting begomovirus pathogenesis is unknown. Regardless, the identification of these proteins and the presence of common DNA binding motifs upstream of the coding regions for those proteins is consistent with existing works, and it has opened up numerous lines of future investigation into whitefly–plant–virus interactions.

## 4. Materials and Methods

### 4.1. Plant Growth, Whitefly Colony Establishment, Feeding, and Sample Collection

Tomato seeds (*Solanum lycopersicum* ‘Florida Lanai’) were germinated under greenhouse conditions maintained at 24–29 °C in flat trays (BWI Apopka, Catalog Number GPPF72S7X) filled with Sun Gro Horticulture soil (Metro-mix 830, BWI Apopka, Apopka, FL, USA, Cat# TX830). Two weeks post emergence, seedlings were transplanted to 4” pots using the same soil and transferred to a Conviron walk-in growth chamber (CMP6060) for the remainder of the experiment. Conviron conditions were as follows: 14 h/10 h light/dark cycle employing fluorescent lights (approximately 1000 ftc at canopy height), maintained at 28 °C, and weekly fertilization (NPK, 20-20-20). To prevent cross-contamination, tomato plants were confined to small mesh insect cages at all times (BioQuip #1450NS68). Four weeks after transplanting, 40 newly emerged (1–3 days post emergence) adult whiteflies (*B. tabaci* MEAM1) were gently aspirated from one of two colonies, either virus-free or ToMoV-infected, and transferred to a clip cage that had been placed on the 4th true leaf of each tomato plant as previously described [96]. The virus-free colony of whiteflies were reared on a non-virus host plant cabbage (*Brassica oleracea* ‘Earliana’), while viruliferous whiteflies were reared on ToMoV-infected tomato (Florida Lanai). For all plants in this study, whitefly feeding was halted after 3 days of leaf access (dpf) by the gentle removal of clip cages and whitefly termination using insecticidal soap (1% potassium salts of fatty acids, Garden Safe, Middleton, WI, USA). For samples at the SOF, the tomato leaf bound within the clip cages was immediately removed and snap frozen for protein extraction. For the samples taken from systemic leaves, the plants continued growing for 7 additional days (10 dpf) after clip cage removal and whitefly termination, at which point the ninth leaf was excised and snap frozen. Plants used for collection at 3 dpf were not the same as those used for the collection of systemic leaves 10 dpf. For leaves collected at the SOF and systemically, we also included a set of control plants whose leaves were subjected to identical clip cage and insecticidal soap applications, but without whitefly feeding. Therefore, our experiment consists of tomato leaves subject to non-viruliferous (+WF) whitefly feeding, viruliferous (+WF^V^) whitefly feeding, and a no-feeding control (NFC), with leaves sampled from each group at the SOF (4th true leaf, 3 dpf) and from systemic leaves (9th true leaf, 10 dpf). The presence of ToMoV in all infected plants was confirmed via Nanopore sequencing. Briefly, tomato genomic DNA was extracted from five systemic leaf samples using the PureGene tissue DNA isolation kit (product # 158667; QIAGEN, Valencia, CA, USA), following the manufacturer’s protocol and stored at −80 °C until needed. Library preparation was performed using the Rapid Sequencing Kit RBK004 protocol (Oxford Nanopore Technologies, Oxford, UK) and loaded onto a 9.4.1 flow cell in a MinION connected to a MinIT with live base calling enabled. The resulting sequencing reads for each sample were mapped to both ToMoV A and B component (NC_001939.1 and NC_001938.1, respectively) sequences.

### 4.2. Proteomics Sample Preparation

Prior to protein extraction, frozen leaf tissue was homogenized by bead beating for three rounds of 30 s intervals at 1500 strokes/minute (Spex SamplePrep, Cat# 1600 MiniG, Metuchen, NJ, USA) with cooling between each cycle on liquid nitrogen. Protein from homogenized tomato leaf tissue was extracted using the previously described MPLex technique [97]. Briefly, pulverized leaf tissue was resuspended in ice cold H_2_O and transferred to a chloroform safe tube. Ice cold methanol was added to the suspension to a methanol/water ratio of 4:3. Samples were vortexed twice for 60 s in a cold room. Then, chloroform was added to the solution to a final chloroform/methanol/H_2_O ratio of 8:4:3. Samples were again vortexed twice for 60 s each. Then, each sample was centrifuged at 5000× *g* for 10 min at 4 °C. The resulting aqueous and organic layers were removed and discarded, leaving the interphase protein precipitate. The protein pellet was washed twice with 1.5 mL ice cold methanol and transferred to a clean tube. After the removal of residual methanol via SpeedVac, each pellet was resuspended in a 50 mM NH_4_HCO_3_ solution containing 8 M urea, and the protein concentration was determined via bicinchoninic acid assay (BCA, Thermo Scientific, Waltham, MA, USA, Cat# 23225). Then, each sample was diluted 8-fold with NH_4_HCO_3_ containing 1.14 mM CaCl_2_ to a final concentration of 1 mM CaCl_2_, followed by the addition of trypsin to a 1:50 (*w*:*w*) trypsin to protein ratio and incubation according to manufacturer’s protocols. Peptides were recovered using C-18 SPE columns (Phenomenex, Torrance, CA, USA, Cat#8B-S001-DAK) and concentrated in a speed vac. Peptides were again quantified by BCA, and a final aliquot of each sample was prepared at 0.1 μg/μL for mass-spectrometry.

### 4.3. Mass Spectrometry, Spectral Search, and Data Analysis

Five μL of each sample was injected into Waters nanoAcquity and separated for 100 min at 300 nl/min on an in-house prepared 70 cm × 75 µm i.d. 3-µm Jupiter C18 column in-line with an LTQ Velos Orbitrap using electrospray ionization. The RAW files were processed via MaxQuant [98] using a FASTA file containing protein sequences of *S. lycopersicum* (via Uniprot [99]), ToMoV DNA A (NC_001939.1), and DNA B (NC_001938.1). The raw data have been submitted to the ProteomeXchange (http://www.proteomexchange.org, identifier PXD019197) [100]. With few exceptions, the default MaxQuant spectral search parameters were left, and they included the variable oxidation of methionine and N-termini as well as a fixed carbamidomethylation with a maximum number of 5 modifications per peptide. A match between a run window of 1 min, 1% protein FDR, and iBAQ protein abundance estimation was also used. The resulting data were imported into Perseus [101] (v1.6.5.0) for further analysis. Our dataset was further filtered by including only proteins identified by ≥2 unique peptides. Furthermore, only proteins that occurred in all 4 biological replicates of at least one treatment were considered (e.g., all 4 samples of local NTC leaves, or all 4 samples of systemic +WF leaves, etc.). The data were log_2_ transformed followed by missing value imputation from a normal distribution with a downshift of 1.8 [101] and median subtraction. Local and systemic leaves differed in age; so, to prevent discovering proteins whose abundance changes naturally during leaf development, we separated local/mature 3 dpf leaf data from younger/systemic 10 dpf data prior to multi-sample statistical analysis (e.g., ANOVA and PCA). For both local and systemic tissue analysis, an ANOVA was performed with a Benjamini–Hochberg [102] (B-H) *p*-value correction (5% FDR). Additional post-hoc analyses included pairwise t-tests with B-H *p*-value corrections (5% FDR). Hierarchical clustering analysis (HCA) was performed using Pearson correlation and average linkage following a standard z-score transformation (z = χ−μ/σ) [101]. Only proteins passing the B-H adjusted ANOVA significance threshold were used in the HCA. The optimal cluster number was determined by a Multi-experiment viewer (MeV) [103] (v.4.9.0) figure of merit [104]. When used, PANTHER (version 14.1 released 14 February 2020) over-representation analysis included a Fisher test and Bonferroni multiple hypothesis test correction using the *S. lycopersicum* genome as a background [35].

### 4.4. Promoter Analysis

An assessment of regulatory motifs was undertaken for the promoters of the top 20 genes whose proteins were observed to have the largest increase and decrease in abundance as chosen based on the pairwise post-hoc comparison of +WF^V^ and +WF in systemic leaves 10 dpf (B-H adj. *q*-value < 0.05). For each of the 20 genes, 2000 base pairs of DNA upstream of the coding region were acquired using Ensembl Plants (release 47) and submitted to Meme-suite Motif Discovery (v5.1.1) [105] using default search settings. Then, each of the motifs discovered was submitted to Gene Ontology for Motifs (GOMo) using the model Arabidopsis and default settings [105]. Promoter sequences for each gene, as well as the GOMo enrichment analysis results for each motif, can be found in Supplemental Table S1.

## 5. Conclusions

Our study has identified hundreds of tomato proteins that differentially accumulate locally and systemically in response to feeding by whiteflies alone and/or whitefly-transmitted ToMoV. At 3 days post feeding (dpf), tomato’s response to ToMoV was small relative to the impact of whitefly feeding and included proteins associated with translation initiation and elongation as well as plasmodesmata dynamics. In contrast, systemic impacts of ToMoV on younger leaves at 10 dpf were more pronounced and included a virus-specific change in plant proteins associated with mRNA maturation and export, RNA-dependent DNA methylation, and other antiviral plant processes. Our experimental observations are largely consistent with previous studies and also identified many novel tomato responses to whitefly feeding and ToMoV infection. 

## Figures and Tables

**Table 1:** Select gene ontology (GO) terms enriched among hierarchical clustering analysis (HCA) clusters.

Tissue	Clust	Select GO Terms (GO Term)	# of Proteins	F.E.	B. adj. *p* Value
Local	1	demethylation (GO:0070988)	2	32.77	2.16 × 10^−3^
	1	mRNA splice site selection (GO:0006376)	1	25.49	4.26 × 10^−2^
	1	defense response (GO:0006952)	3	6.37	1.26 × 10^−2^
	1	translational elongation (GO:0006414)	5	3.39	1.72 × 10^−2^
	2	chaperone-mediated protein folding (GO:0061077)	3	7.81	7.60 × 10^−3^
	2	translational elongation (GO:0006414)	7	3.29	6.26 × 10^−3^
	2	mRNA processing (GO:0006397)	4	3.21	3.85 × 10^−2^
	2	proteolysis (GO:0006508)	10	2.77	3.95 × 10^−3^
Systemic	1	Golgi to vacuole transport (GO:0006896)	1	66.87	1.59 × 10^−2^
	1	regulation of actin polymerization or depolymerization (GO:0008064)	1	44.58	2.32 × 10^−2^
	1	chromatin silencing (GO:0006342)	2	36.01	1.53 × 10^−3^
	2	RNA metabolic process (GO:0016070)	5	3.51	1.32 × 10^−2^
	3	Golgi to plasma membrane transport (GO:0006893)	1	37.49	2.76 × 10^−2^
	3	defense response to bacterium (GO:0042742)	1	22.49	4.47 × 10^−2^
	3	proteolysis (GO:0006508)	3	4.11	3.67 × 10^−2^
	4	DNA demethylation (GO:0070988)	2	38.96	1.54 × 10^−3^
	4	cellular response to oxidative stress (GO:0034599)	4	34.09	9.14 × 10^−6^
	4	protein ubiquitination (GO:0016567)	5	7.14	7.89 × 10^−4^
	4	DNA repair (GO:0006281)	3	3.97	4.15 × 10^−2^
	4	cellular response to stimulus (GO:0051716)	8	2.88	7.25 × 10^−3^
	5	glycolytic process (GO:0006096)	4	83.87	2.62 × 10^−7^
	5	cellular amino acid biosynthetic process (GO:0008652)	6	37.47	1.96 × 10^−8^
	5	protein transmembrane import into intracellular organelle (GO:0044743)	3	37.47	8.65 × 10^−5^

B., Bonferroni; F.E., fold enrichment; #, number.

**Table 2:** Motif discovery and ontology enrichment analysis of similar motifs upstream of genes in Arabidopsis.

Meme Motif Discovery				GOMo Motif Ontology Enrichment		
R.	Motif	*E*-Val	Sites	GO Term	*q*-Val	GO Name
Increased (+WF^V^v. +WF)	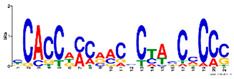			GO:0009507	1 × 10^−3^	Chloroplast ^1^
				GO:0009941	1 × 10^−2^	Chloroplast envelope ^1^
		3 × 10^−7^	13	GO:0003700	1 × 10^−2^	Transcription factor activity ^2^
				GO:0006355	2 × 10^−2^	Transcription regulation, DNA-dependent ^3^
				GO:0003677	3 × 10^−2^	DNA binding ^2^
	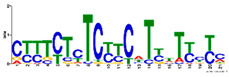			GO:0003700	9 × 10^−5^	Transcription factor activity ^2^
				GO:0048366	4 × 10^−4^	Leaf development ^3^
		4 × 10^−4^	20	GO:0007623	5 × 10^−3^	Circadian rhythm ^3^
				GO:0004842	5 × 10^−3^	Ubiquitin–protein ligase activity ^2^
				GO:0010599	4 × 10^−2^	Production of lsiRNA involved in RNAi ^3^
Decreased (+WF^V^ v. +WF)	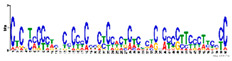			GO:0009507	2 × 10^−4^	Chloroplast ^1^
				GO:0003700	2 × 10^−4^	Transcription factor activity ^2^
		6 × 10^−24^	10	GO:0003677	2 × 10^−4^	DNA binding ^2^
				GO:0048366	4 × 10^−2^	Leaf development ^3^
				GO:0009941	4 × 10^−2^	Chloroplast envelope ^1^
	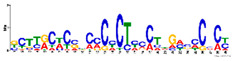					
				GO:0009507	1 × 10^−3^	Chloroplast ^1^
		6 × 10^−10^	14	GO:0003700	2 × 10^−3^	Transcription factor activity ^2^
				
				
	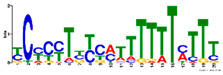			GO:0003700	2 × 10^−4^	Transcription factor activity ^2^
				GO:0006355	2 × 10^−3^	Transcription regulation, DNA-dependent ^3^
		2 × 10^−2^	20	GO:0009414	5 × 10^−3^	Response to water deprivation ^3^
				GO:0009956	2 × 10^−2^	Radial pattern formation ^3^
				GO:0009753	4 × 10^−2^	Response to jasmonic acid stimulus ^3^

R., Regulation at 10 dpf in systemic leaves; Sites, the number of motif occurrences within the 20 tested promoter regions; *E*-val, *E*-value; *q*-val, *q*-value, ^1^ CC; ^2^ MF; ^3^ BP.

## Supplementary Materials

Supplementary materials can be found at https://www.mdpi.com/1422-0067/21/19/7241/s1. Table S1. Main data analysis file containing IDs, abundance values before and after normalization, ANOVA and pairwise post-hoc statistical analyses, as well as gene ontology enrichment results from each cluster and GOMo promoter analysis.

## Abbreviations

Dpf	Days Post Feeding
GO	Gene Ontology
HCA	Hierarchical Cluster Analysis
NFC	No Feeding Control
PTGS	Post-Translational Gene Silencing
RdDM	RNA-Dependent DNA Methylation
SG	Stress Granule
SOF	Site of Feeding
TGS	Transcriptional Gene Silencing
ToMoV	Tomato Mottle Virus
WF	Whitefly
